# COVID-19-specific transcriptomic signature detectable in blood across multiple cohorts

**DOI:** 10.3389/fgene.2022.929887

**Published:** 2022-08-05

**Authors:** Tommi Välikangas, Sini Junttila, Kalle T. Rytkönen, Anu Kukkonen-Macchi, Tomi Suomi, Laura L. Elo

**Affiliations:** ^1^ Turku Bioscience Centre, University of Turku and Åbo Akademi University, Turku, Finland; ^2^ Institute of Biomedicine, University of Turku, Turku, Finland

**Keywords:** COVID-19, signature, RNA-seq, single-cell, infection, severity

## Abstract

The coronavirus disease 2019 (COVID-19) caused by the severe acute respiratory syndrome coronavirus 2 (SARS-CoV-2) is spreading across the world despite vast global vaccination efforts. Consequently, many studies have looked for potential human host factors and immune mechanisms associated with the disease. However, most studies have focused on comparing COVID-19 patients to healthy controls, while fewer have elucidated the specific host factors distinguishing COVID-19 from other infections. To discover genes specifically related to COVID-19, we reanalyzed transcriptome data from nine independent cohort studies, covering multiple infections, including COVID-19, influenza, seasonal coronaviruses, and bacterial pneumonia. The identified COVID-19-specific signature consisted of 149 genes, involving many signals previously associated with the disease, such as induction of a strong immunoglobulin response and hemostasis, as well as dysregulation of cell cycle-related processes. Additionally, potential new gene candidates related to COVID-19 were discovered. To facilitate exploration of the signature with respect to disease severity, disease progression, and different cell types, we also offer an online tool for easy visualization of the selected genes across multiple datasets at both bulk and single-cell levels.

## Introduction

The coronavirus disease 2019 (COVID-19) caused by the severe acute respiratory syndrome coronavirus 2 (SARS-CoV-2) has flared into a worldwide pandemic. Despite ongoing massive vaccination efforts, the disease is still actively spreading in many parts of the world. Although a large proportion of the SARS-CoV-2-infected individuals remain asymptomatic or experience only mild symptoms, an estimated 6%–15% of them undergo severe symptoms (Guan et al., 2020; Wu and McGoogan, 2020). The case fatality rate is estimated to be from 1.2% to 2.6% (Russell et al., 2020; Verity et al., 2020; Wu and McGoogan, 2020), with severe and fatal outcomes substantially more pronounced in older patients (Verity et al., 2020; Wu and McGoogan, 2020; Yang et al., 2020). Severe forms of the disease are often associated with a hyperinflammatory state, the so-called cytokine storm, where increased levels of many proinflammatory cytokines and lymphopenia have been observed (Huang and Pranata, 2020; Ong et al., 2020; Pedersen and Ho, 2020). Overall, the high mortality in COVID-19 is a consequence of alveolar damage and pneumonia, cardiovascular complications, and multi-organ failure (Brunetta et al., 2021).

Several studies have sought to elucidate the human host factors and immune mechanisms related to COVID-19, its severity, and post-infection recovery. Beyond studies of the respiratory microenvironment, a number of bulk and single-cell RNA-sequencing (RNA-seq) studies have focused on responses observed in the periphery using whole blood (Ng et al., 2021), peripheral blood mononuclear cells (PBMCs) (Lee et al., 2020; Wilk et al., 2020; Liu et al., 2021), or isolated subsets of blood cells (Brunetta et al., 2021; Overmyer et al., 2021). However, most of these studies have focused on comparing the transcriptome profiles of COVID-19 patients with healthy controls (e.g., Lee et al., 2020; Liu et al., 2021), while much fewer studies have compared the COVID-19 signatures with other common respiratory infections, such as influenza (Lee et al., 2020; McClain et al., 2021; Ng et al., 2021).

To establish a robust transcriptomic signature specific to COVID-19 and to deepen the understanding of the disease-related host processes related specifically to COVID-19, we reanalyzed transcriptome data from nine independent cohort studies (Arunachalam et al., 2020; Lee et al., 2020; Wilk et al., 2020; Brunetta et al., 2021; Combes et al., 2021; Liu et al., 2021; McClain et al., 2021; Ng et al., 2021; Overmyer et al., 2021), covering over 500 individual profiles, including patients with common respiratory infections (influenza, seasonal coronavirus, and bacterial pneumonia) together with COVID-19 patients and healthy controls. We discovered a COVID-19 specific signature that appeared systematically across the cohorts. Additionally, we explored the association of the signature genes with disease severity, disease progression, and different cell types. To facilitate easy investigation of the signature, we also present an online tool for easy visualization of the selected genes across multiple datasets at both bulk and single-cell levels (https://elolab.shinyapps.io/COVID19).

## Results

To establish a robust COVID-19 specific transcriptomic signature, we analyzed a total of nine previously published COVID-19 bulk or single-cell RNA-seq datasets, containing a total of 511 individuals (Table 1). Two of the datasets (Lee et al., 2020; McClain et al., 2021) were used for signature identification, while the other seven datasets (Arunachalam et al., 2020; Wilk et al., 2020; Brunetta et al., 2021; Combes et al., 2021; Liu et al., 2021; Ng et al., 2021; Overmyer et al., 2021) were used for validating the signal. All of the datasets were preprocessed as similarly as possible and the reproducibility optimized test statistic (ROTS) was used to detect the COVID-19 specific signal (Seyednasrollah et al., 2016; Suomi et al., 2017). To facilitate further use of the data and the results, we also compiled all the datasets and the associated clinical and other information as an online resource for visualizing the COVID-19 specific transcriptomic signatures. The tool is freely available at https://elolab.shinyapps.io/COVID19/.

**TABLE 1:** Datasets used in this study.

Name	Sample type	Number of samples	Cohort	Method	Accession	References
McClain	Whole blood	46 COVID-19	US	RNA-seq	GSE161731	McClain et al. (2021)
		59 seasonal coronavirus				
		17 influenza				
		20 bacterial pneumonia				
		19 healthy				
Lee	PBMC	11 COVID-19	South Korea	scRNA-seq	GSE149689	Lee et al. (2020)
		5 influenza				
		4 healthy				
Ng	Whole blood	7 COVID-19	US	RNA-seq	GSE163151	Ng et al. (2021)
		20 influenza				
		6 bacterial sepsis				
		20 healthy				
Combes	Whole blood	21 COVID-19	US	scRNA-seq	GSE163668	Combes et al. (2021)
		11 non-COVID				
		14 healthy				
Overmyer	Leukocytes	102 COVID-19	US	RNA-seq	GSE157103	Overmyer et al. (2021)
		26 non-COVID				
Arunachalam	PBMC	17 COVID-19	US	RNA-seq	GSE152418	Arunachalam et al. (2020)
		17 healthy				
Liu	PBMC	33 COVID-19	China	CITE-seq	GSE161918	Liu et al. (2021)
		14 healthy				
Wilk	PBMC	7 COVID-19	US	scRNA-seq	GSE150728	Wilk et al. (2020)
		6 healthy				
Brunetta	Monocytes	6 COVID-19	Italy	RNA-seq	GSE160351	Brunetta et al. (2021)
		3 healthy				

### COVID-19-specific transcriptomic signature of 149 genes appears systematically across multiple cohorts

**FIGURE 1:**
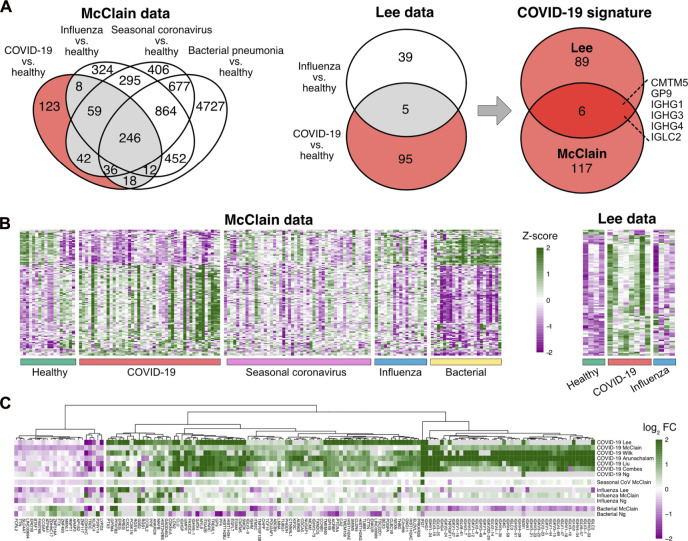
Determination of the COVID-19 specific signature **(A)** Differentially expressed genes in COVID-19 and other infections compared to healthy controls in the McClain and Lee data. Genes that were differentially expressed specifically in the COVID-19 patients compared to healthy controls and not between other disease states (influenza, bacterial pneumonia, seasonal coronavirus) and healthy controls are shown with a red background. The Venn diagram on the right shows the number of shared COVID-19 specific differentially expressed genes in the McClain and Lee data. **(B)** Heatmaps of the expression levels of COVID-19 specific genes detected from the McClain and Lee data. **(C)** Fold changes of the signature genes in COVID-19 and other infections compared to healthy controls across multiple independent cohorts.

To discover a robust set of genes related specifically to COVID-19 in peripheral blood, we considered two datasets, covering COVID-19 patients, patients with other common respiratory infections, as well as healthy controls: the McClain data are a whole blood RNA-seq dataset (McClain et al., 2021), while the Lee data are a single-cell RNA-seq dataset of PBMCs (Lee et al., 2020). To focus on COVID-19 specific signals, we identified those genes that were differentially expressed between COVID-19 patients and healthy controls at a false discovery rate (FDR) of 0.05 but not between any other disease state (influenza, seasonal coronavirus, and bacterial pneumonia) and healthy controls. This analysis suggested altogether 212 COVID-19 specific genes from the two datasets (Figures 1A,B). Out of these genes, 123 were discovered from the whole blood McClain data and 95 from the PBMC Lee data, with an overlap of six findings between the datasets: IGHG1, IGHG3, IGHG4, IGCL2, CMTM5, and GP9.

To further refine and validate our COVID-19 specific set of genes, we used six additional independent datasets, including both bulk and single-cell RNA-seq data from whole blood (Combes et al., 2021; Ng et al., 2021), PBMCs (Arunachalam et al., 2020; Wilk et al., 2020; Liu et al., 2021), or leukocytes (Overmyer et al., 2021). Of the 212 genes, altogether, 149 genes were consistently changed across the datasets (Figure 1C; Supplementary Table S1). Majority of these genes (85%) were up-regulated in COVID-19 when compared to the healthy controls.

### COVID-19-specific signature is dominated by immunoglobulin-related genes

Our COVID-19 specific signature was dominated by immunoglobulin-related genes, including several immunoglobulin heavy chain variable (IGHV), immunoglobulin lambda variable (IGLV), immunoglobulin lambda constant (IGLC), immunoglobulin heavy constant gamma (IGHG), and immunoglobulin kappa variable (IGKV) region genes, among others. In particular, four of the shared six genes between the McClain and Lee data were immunoglobulin encoding genes: IGHG1, IGHG3, IGHG4, and IGLC2, all of which were consistently upregulated in a COVID-19 specific manner across the datasets. Similarly, other immunoglobulin-related genes were consistently upregulated in COVID-19 across the datasets, such as pentraxin 3 (PTX3), which has earlier been associated with COVID-19 (Brunetta et al., 2021).

The functional enrichment results among the COVID-19 specific genes were also dominated by the immunoglobulin-related signal (Table 2, Supplementary Table S2), the most enriched processes being the classical pathway of complement activation (GO:0006958, hypergeometric test, FDR < 10^−53^) and immunoglobulin production (GO:0002377, FDR < 10^−23^). Among the more specific terms, Fc receptor-related processes were enriched, such as the Fc-gamma receptor signaling pathway (GO:0038094, FDR < 10^−34^). Overall, the detected immunoglobulin signal was strong and consistent across the datasets. While this immunoglobulin signal appeared to be specifically related to COVID-19 in the McClain data, the involved genes were not expressed highly enough in the Lee and Ng datasets including, other infections, to confirm this specificity.

**TABLE 2:** Functionally enriched gene sets among the COVID-19 specific signature. Summary gene sets at false discovery rate (FDR) of 0.05 are shown.

Summary gene set	Gene set ID	Source of gene set	FDR
Complement activation, classical pathway	GO:0006958	GO biological processes	<10^−53^
Immunoglobulin production	GO:0002377	GO biological processes	<10^−23^
RUNX1 regulates genes involved in megakaryocyte differentiation and platelet function	R-HSA-8936459	Reactome gene sets	<10^−5^
Antimicrobial humoral response	GO:0019730	GO biological processes	<10^−4^
Platelet activation	GO:0030168	GO biological processes	<10^−4^
Mitotic nuclear division	GO:0140014	GO biological processes	<0.01
Multicellular organismal homeostasis	GO:0048871	GO biological processes	0.02
Positive regulation of fibroblast proliferation	GO:0048146	GO biological processes	0.02

### COVID-19-specific signature involves induction of hemostasis

Another strongly induced signal in the COVID-19 specific signature was related to hemostasis, including significant enrichment of platelet activation (GO:0030168, FDR < 10^−4^) (Table 2, Supplementary Table S2). Two of the shared-six genes between the McClain and Lee data were related to platelet function: glycoprotein IX platelet (GP9) and CKLF-like MARVEL transmembrane domain-containing protein 5 (CMTM5), both of which were consistently upregulated in a COVID-19 specific manner across the datasets. GP9 is a small membrane glycoprotein localized on human platelets and is associated with hemostasis and platelet adhesion to blood vessels in injured vascular surfaces (Resource Coordinators et al., 2017), whereas CMTM5 has been associated with platelet function in response to aspirin and is related to cardiovascular outcomes (Voora et al., 2013).

Interestingly, the COVID-19 specific signature was also highly enriched with genes from the Reactome pathway “RUNX1 regulates genes involved in megakaryocyte differentiation and platelet function” (R-HSA-8936459, FDR < 10^−5^), showing consistent COVID-19 specific upregulation. Megakaryocytes are large bone marrow cells responsible for the production of platelets (Choi et al., 1995), while the runt-related transcription factor 1 (RUNX1) is considered a master regulator in hematopoiesis, involved in the maturation of hematopoietic stem cells into mature blood cells (Okuda et al., 2001).

### Several cell cycle-related genes are dysregulated in COVID-19

Finally, we observed enrichment of the cell cycle and mitotic division-related processes among our COVID-19 specific genes (Table 2, Supplementary Table S2). Many of the cell cycle-related genes, such as aurora kinase B (AURKB) and cyclin-dependent kinase inhibitor 1A (CDKN1A, p21), were consistently upregulated in a COVID-19 specific manner, while some were consistently downregulated, such as cyclin-dependent kinase inhibitor 1C (CDKN1C, p57). Coronaviruses in general have been shown to manipulate the cell cycle of host cells, especially the arrest of the cell cycle at specific cell cycle checkpoints (Dove et al., 2006; Simabuco et al., 2020; Su et al., 2020) and also mitotic events (Bock and Ortea, 2020). In agreement, several distinct terms related to different cell cycle phases, such as mitotic prometaphase (R-HSA-68877, FDR = 0.02), mitotic sister chromatid segregation (GO:0000070, FDR < 0.01), and mitotic nuclear division (GO:0140014, FDR < 0.01) were enriched among our COVID-19 specific signature (Supplementary Table S2).

### COVID-19-specific signature is associated with disease severity

Next, we investigated the association of our COVID-19 specific signature with the severity of the disease using those six datasets (McClain, Lee, Arunachalam, Combes, Liu, and Overmeyer) that had severity information available. The patients were divided into two categories based on whether they required mechanical ventilation or intensive care (severe) or not (mild) according to the original studies. All the patients in both categories were hospitalized apart from patients in the mild category in the McClain data.

**FIGURE 2:**
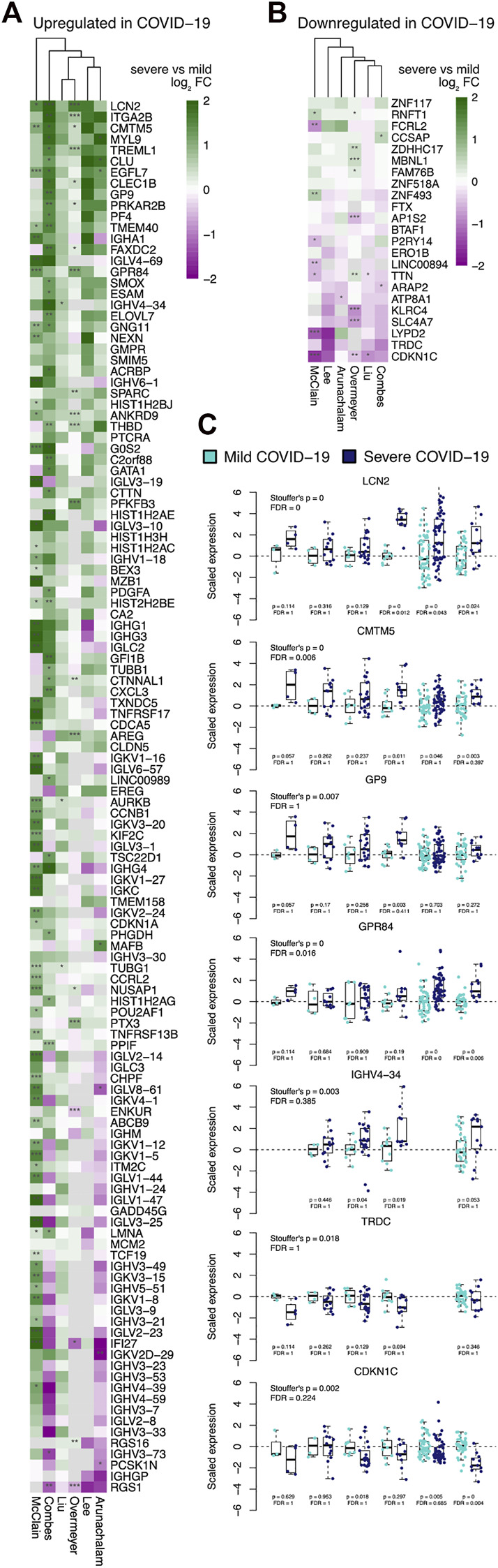
Association of the COVID-19 specific signature genes with disease severity heatmaps showing logarithmic fold changes between the severe and mild cases across the different datasets separately for **(A)** upregulated and **(B)** downregulated COVID-19 specific genes. The significance of Wilcoxon rank-sum test is indicated with asterisks in the heatmaps: *p < 0.05, **p < 0.01, and ***p < 0.001. **(C)** Representative examples of genes with consistently higher or lower expression in the severe disease, with the individual expression values scaled by the average of the mild cases for each data; visualizations of all signature genes are available in our online tool (https://elolab.shinyapps.io/COVID19/).

Many of the COVID-19 specific upregulated genes tended to have higher expression in severe diseases compared to milder diseases, while considerable variation was observed between the datasets (Figure 2A). Likewise, many of the COVID-19 specific downregulated genes tended to have lower expression in severe diseases compared to milder diseases (Figure 2B). Especially the hemostasis-related genes were generally and consistently upregulated in the severe cases, including the shared gene findings between the McClain and Lee data; GP9 and CMTM5 (Figures 2A,C). The immunoglobulin encoding genes, on the other hand, showed considerable variation between the datasets, with the exception of IGHV4-34, which was systematically upregulated in the severe cases in all four datasets where it was detected (Figures 2A,C).

To investigate whether specific functions were related to disease severity, we explored functional enrichment among those 52 genes that were consistently up-regulated in the severe disease when compared to the milder disease across the datasets (Figure 2A). The most enriched functional terms were related to RUNX1 (“RUNX1 regulates genes involved in megakaryocyte differentiation and platelet function,” R-HSA-8936459, FDR < 10^−8^), hemostasis (R-HSA-109582, FDR < 10^−6^), platelet activation, signaling and aggregation (R-HSA-76002, FDR < 0.001), and blood coagulation (GO:0007596, FDR < 0.001), suggesting dysregulation related to platelet function and blood clotting in patients with more severe disease (Supplementary Table S2).

### COVID-19-specific signature is not generally associated with time from symptom onset

In addition to disease severity, we investigated the association of our COVID-19 specific signature genes with time from symptom onset using those four datasets (Arunachalam, Combes, Liu, and Wilk) that had the symptom onset information available. The associations were determined using Pearson correlation between the reported number of days from the symptom onset and the measured gene expression level, scaled by the corresponding control average.

**FIGURE 3:**
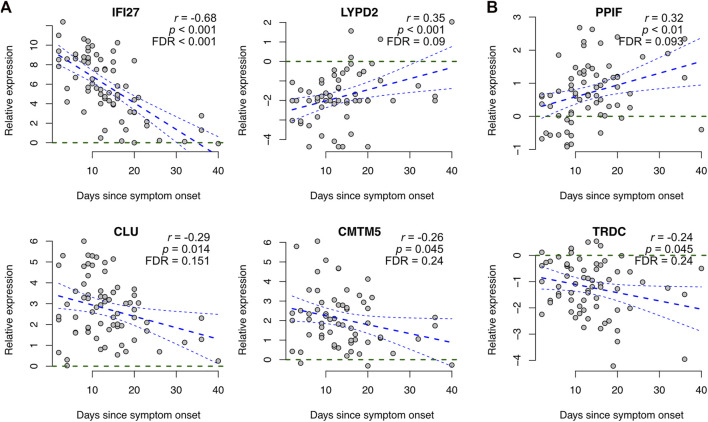
Association of COVID-19 specific signature genes with time from symptom onset. Representative examples of genes showing a significant association between expression and time from symptom onset, including **(A)** genes becoming closer to the healthy controls at later stages and **(B)** genes showing larger differences at the later stages. Gene expression levels of COVID-19 patients were scaled to healthy controls within each dataset and plotted as a function of days since symptom onset across all datasets that had the symptom onset information available (Arunachalam, Combes, Liu, and Wilk), including linear regression with 95% confidence interval. Additionally, the Pearson correlation coefficients (r) and the corresponding *p*-values are shown. Visualizations of all signature genes are available in our online tool (https://elolab.shinyapps.io/COVID19/).

Most (∼80%) of the signature genes did not show a significant correlation with the time from symptom onset (*p* > 0.05, Supplementary Table S3). Among the genes showing a trend (*p* < 0.05), most of them had larger expression changes at the early stages of the disease than at the later stages, at which the expression levels typically become closer to the healthy controls again (Figure 3A). However, two exceptions stood out: for peptidylprolyl isomerase F (PPIF) and T cell receptor delta constant (TRDC) the differences tended to be larger at the later stages (Figure 3B).

### COVID-19-specific transcriptional signal comes from multiple cell types

**FIGURE 4:**
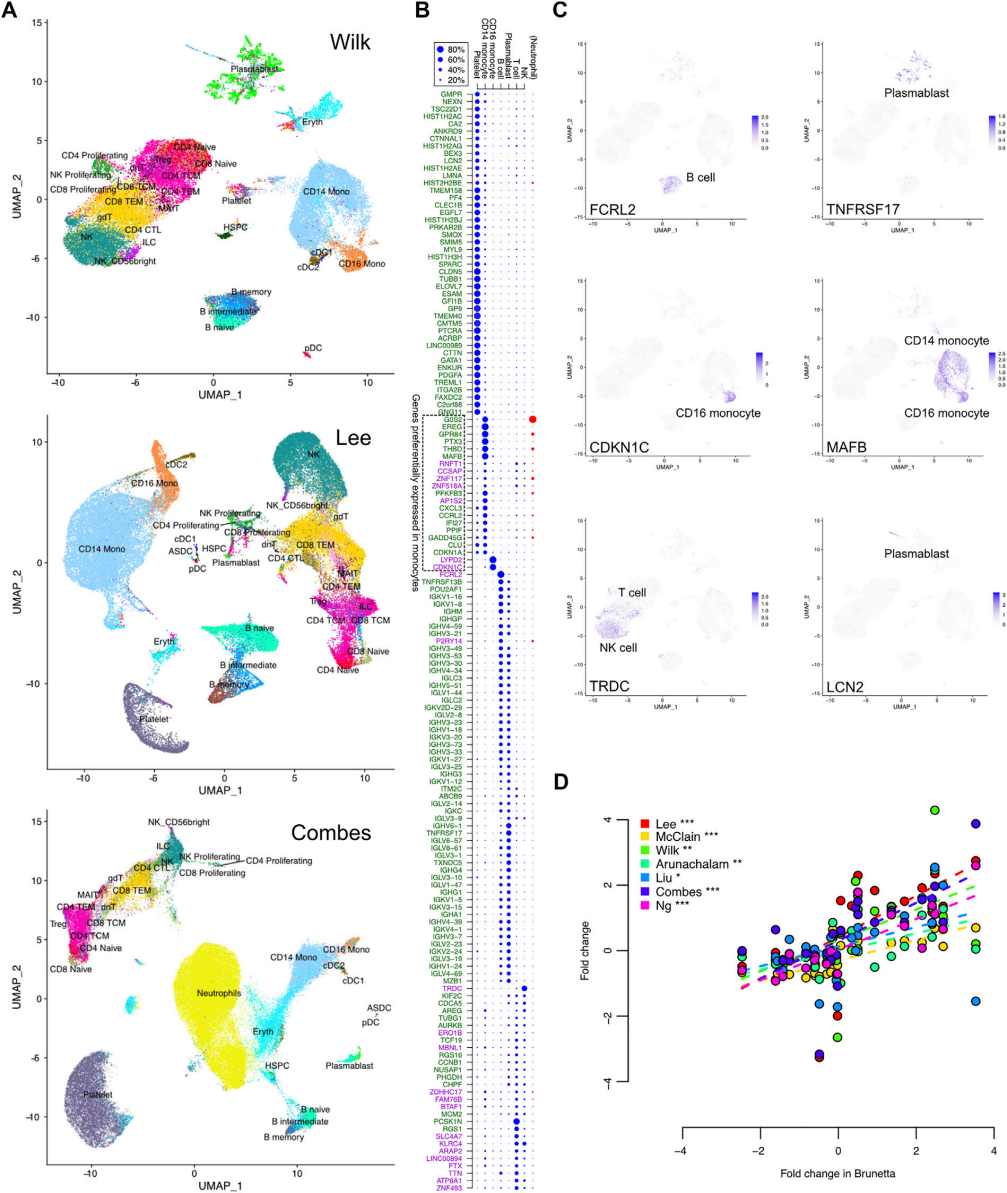
COVID-19 specific expression signature in single-cell RNA-seq data. **(A)** Uniform manifold approximation and projection (UMAP) clustering of the Wilk, Lee, and Combes single-cell RNA-seq datasets together with harmonized cell type annotations. **(B)** Contribution of the different cell types to the observed bulk expression of the signature genes, as measured by the relative proportions of sequencing reads assigned to the different cell subsets that are common across the datasets. Neutrophil proportion was available only in the Combes data and is shown separately. The upregulated and downregulated COVID-19 specific genes are indicated by the green and purple text, respectively. **(C)** Expression of selected representative genes in the single cells of the Wilk dataset; visualizations of all signature genes are available in our online tool (https://elolab.shinyapps.io/COVID19/). These include FCRL2 and TNFRSF17 as examples of responses in B cells and blasmablasts, CDKN1C in CD16 monocytes as an example of cell cycle regulation, and LCN2 as an example of a novel marker from a cell population of developing neutrophils defined by Wilk. **(D)** Comparison of logarithmic fold changes between COVID-19 cases and healthy controls for genes preferentially expressed in monocytes, as indicated in panel **(B)**. The data are from seven bulk expression datasets (*y*-axis) and isolated monocytes (*x*-axis, Brunetta data). The significance of Pearson correlation is indicated with asterisks: *p < 0.05, **p < 0.01, ***p < 0.001.

To further investigate the source of the COVID-19 specific expression signal, we analyzed the signature genes at the single-cell level using three single-cell RNA-seq datasets (Wilk, Lee, and Combes) with harmonized cell type annotations (Hao et al., 2021) (Figure 4A).

In general, there were more platelets in the COVID-19 cases than in the healthy controls (Supplementary Table S4). In particular, in the whole-blood Combes data, ∼17% of the cells in COVID-19 cases were platelets, while the proportion was on average 9% in the healthy controls (Wilcoxon test *p* < 0.05). Similarly, there were significantly more neutrophils in the COVID-19 cases than in the controls in the Combes data (49% vs. 21%, *p* < 0.0001). When considering only the PBMC cell types, the COVID-19 cases had more plasmablasts in the Wilk and Combes data (on average 9% and 2%, respectively) than the controls (0.5% or below in both datasets, *p* < 0.01), whereas in the Lee data, both groups had less than 0.5% of the cells classified as plasmablasts. There was also a systematic tendency to have larger proportions of CD14 monocytes in COVID-19 (on average 30%–38%) than in controls (on average 15%–23%, *p* < 0.001 in Combes, *p* < 0.1 in Wilk, *p* = 0.2 in Lee data, Supplementary Table S4).

Next, we investigated which cell types contributed most to the observed bulk expression of the signature genes (Figures 4B,C). This was done by assigning the sequencing reads to different cell subsets and determining their relative proportions. Among the COVID-19 specific signature genes, ∼30% were preferentially expressed in platelets (Figure 4B). All of them were upregulated in the bulk datasets, likely reflecting the increased proportion of platelets in COVID-19 compared to controls. A total of ∼40% of the signature genes were preferentially expressed in B cells or plasmablasts, with most genes expressed in both cell types. These were essentially the immunoglobulin-related genes, which were upregulated in COVID-19 compared to the controls. In total ∼20% of the signature genes were preferentially expressed in T cells or NK cells; however, many of these genes were expressed also in several other cell types. Finally, ∼10% of the COVID-19 specific signature genes were preferentially expressed in CD14 monocytes and ∼1% in CD16 monocytes, including both upregulated and downregulated genes; many of these genes were also expressed in neutrophils in the whole-blood Combes data.

Finally, we confirmed the observed monocyte-related COVID-19 specific signal by comparing the bulk expression levels of the preferentially monocyte-expressed genes to those observed in isolated monocytes (Brunetta data). Indeed, the bulk expression changes of the monocyte-specific genes were very well in line with the changes calculated from the Brunetta monocyte data, with consistent upregulation and downregulation (Figure 4D) (Brunetta et al., 2021), confirming the relevance of the detected signature and potential differences in the monocytes between COVID-19 patients and controls.

### COVID-19-specific signature is associated with multiple known COVID-19-related drugs

Finally, we investigated the associations of the COVID-19 specific signature with known drug and chemical compound signatures using the Library of Integrated Network-based Cellular Signatures (LINCS) database (Koleti et al., 2017; Keenan et al., 2018). Only the most relevant findings were considered when accounting for both the significance (*p* < 0.01) and the concordance (effect size) (concordance value < −0.35) of the connected drugs and chemical compounds. Interestingly, several of the top associated drugs and compounds showing negative concordance have earlier been suggested to be useful against COVID-19 by independent sources and by distinct mechanisms (Table 3, Supplementary Table S5). For instance, the anti-inflammatory theophylline, which is used for the treatment of asthma and chronic obstructive pulmonary disease, has been associated with increased respiratory rate and oxygenation score in COVID-19 pneumonia patients (Wall et al., 2021), and its potential as a relevant candidate to treat COVID-19 patients was recently reviewed based on computational studies (Montaño et al., 2022). Cyclosporin A has been associated with decreased COVID-19 mortality (Guisado-Vasco et al., 2020) and it has been demonstrated to act as an antiviral against SARS-CoV-2 in preclinical infection models (Sauerhering et al., 2022). Fenofibrate has been suggested to enable faster recovery of COVID-19 patients compared to patients treated with standard care (Nahmias et al., 2021). The aminoglycoside antibiotic amikacin has been predicted *in silico* both as a potential inhibitor of the main protease of SARS-CoV-2 (Ahmed et al., 2021) and another enzyme (Elbadwi et al., 2021), as well as a potential inhibitor of the interaction between the SARS-CoV-2 spike protein S1 domain and host ACE2 receptor (Prajapat et al., 2020). The cancer drug lapatinib has been suggested to effectively block SARS-CoV-2 replication in human pulmonary fibroblasts (MRC5 cell line) (Raymonda et al., 2020), while another cancer drug gemcitabine has been shown to block the viral protein expression in virus-infected human lung epithelial cells (Calu-3) (Jang et al., 2021) and kidney epithelial cells (Vero-E6) (Zhang et al., 2020a). The cancer drug sunitinib, on the other hand, has been reported to reduce SARS-CoV-2 infectivity (Wang et al., 2020). Captopril (DrugBank DB01197) and quinapril (DrugBank DB00881) belong to angiotensin-converting enzyme (ACE) inhibitors, which are widely used for the treatment of hypertension and have been associated with reduced risks of COVID-19 (Hippisley-Cox et al., 2020; Tepasse et al., 2022).

**TABLE 3:** Top drug and chemical compound signatures negatively associated with the COVID-19 specific signature. Signatures with *p* < 0.01 and negative concordance value below −0.35 are listed.

Drug/Compound	Signature ID	Source of signature	Concordance	*p*-value
Lapatinib	PG_2820	Pharmacogenomics	−0.42	0.0001
Gemcitabine	PG_2488, PG_2404	Pharmacogenomics	−0.38	0.0005
Sulfadimethoxine	DM_4847	Drug Matrix	−0.55	0.001
Geldanamycin	PG_2042, PG_2102	Pharmacogenomics	−0.36	0.001
Sunitinib	PG_4065	Pharmacogenomics	−0.35	0.001
Amikacin	DM_1692	Drug matrix	−0.52	0.002
Theophylline	DM_4986	Drug matrix	−0.51	0.002
Enoxacin	DM_2916	Drug matrix	−0.51	0.002
Alendronic acid	DM_1630	Drug matrix	−0.50	0.003
Fenofibrate	DM_3102	Drug matrix	−0.50	0.003
Captopril	DM_2148	Drug matrix	−0.49	0.004
Ofloxacin	DM_4225, DM_4227	Drug matrix	−0.48	0.005
Stannous fluoride	DM_4809, DM_4810	Drug matrix	−0.47	0.006
Quinapril	DM_4566	Drug matrix	−0.47	0.006
Cyproheptadine	DM_2616	Drug matrix	−0.46	0.007
Choline chloride	DM_2382	Drug matrix	−0.45	0.009
Cyclosporin A	DM_2594	Drug matrix	−0.44	0.009

Besides confirming the relevance of our COVID-19 specific gene signature, these results also suggest possible new drugs with potential connections to COVID-19. For instance, enoxacin is a broad-spectrum antibiotic that has recently been suggested to also have antiviral activity against various viruses by enhancing RNA interference (RNAi) as an antiviral defense mechanism (Xu et al., 2019; Scroggs et al., 2020). A recent *in silico* analysis suggested the RNA genome of SARS-CoV-2 is a suitable substrate for DICER activity and enoxacin is a promising candidate for COVID-19 treatment (Ahmadi and Moradi, 2021). Similarly, ofloxacin has been suggested to enhance RNAi activity (Zhang et al., 2008). The potential antiviral property of fluoroquinolone antibiotics (such as ofloxacin) against DNA and RNA viruses is well documented (Ikeda et al., 1987; Witvrouw et al., 1998; Dalhoff, 2015). The potential action of fluoroquinolones such as ciprofloxacin, moxifloxacin, and levofloxacin has been demonstrated for the treatment of SARS-CoV-2 associated pneumonia (Karampela and Dalamaga, 2020; Marciniec et al., 2020) and these antibiotics were also recommended to treat community-acquired pneumonia in COVID-19 patients (Metlay and Waterer, 2020). Cyproheptadine (DrugBank DB00434) is a serotonin antagonist. Interestingly, a recent study of *in vivo* platelet activation reported a significant COVID-19 specific increase in plasma serotonin levels compared to healthy controls and patients with acute respiratory distress syndrome without COVID-19 (Zaid et al., 2021). In a case study, COVID-19 patients whose symptoms resembled serotonin syndrome were treated with cyproheptadine (Keith et al., 2021).

### COVID-19 shares several transcriptomic changes with other viral and bacterial infections

While our focus was on host genes related specifically to COVID-19 in peripheral blood, we also investigated functional enrichment of pathways and processes among genes similarly regulated in COVID-19 and other viral or bacterial infections (influenza, seasonal coronavirus, and bacterial pneumonia). Altogether, 246 genes shared differential regulation between all these disease states versus the healthy controls in the McClain data (Figure 1A), the majority of which (89%) were downregulated. Functional enrichment analysis of the shared differentially regulated genes identified the KEGG ribosome pathway as the most distinctively enriched (hsa03010, FDR < 10^−63^, Supplementary Table S2), with almost all of the genes downregulated in the different disease states, suggesting an overall downregulation of the ribosome pathway. Similarly, several other ribosome-related functionalities were enriched, such as the TRBP-containing protein complex involved in microRNA-mediated silencing (CORUM:5380, FDR < 10^−10^), ribonucleoprotein complex subunit organization (GO:0071826, FDR < 10^−9^), and the mitochondrial 55S ribosome (CORUM:320, FDR < 10^−5^).

Furthermore, investigation of the shared differentially regulated genes in COVID-19 and any other disease state (grey area in Figure 1A Venn diagrams), excluding the effect of the 246 common genes regulated across all the disease states, resulted in 180 genes, of which 59% were downregulated. Functional enrichment analysis of these genes did not reveal any strong enrichment (Supplementary Table S2), suggesting variable functions among the host genes. This is in sharp contrast to the COVID-19 specific signature or the genes commonly regulated between all the disease states, for which striking enrichments were discovered. The genes were included in various processes, such as those related to cell death, immunoglobulin production, interferon response, and hemostasis, but none of them remained statistically significant after multiple hypothesis correction. For comparison, processes active specifically in bacterial pneumonia (top 500 most differentially expressed genes in patients with bacterial pneumonia compared to the healthy controls and not detected in any other disease state) were related to the regulation of leukocyte activation (GO:0002694, FDR < 10^−15^), lymphocyte activation (GO:0046649, FDR < 10^−15^), T helper cell 17 (Th17) differentiation (hsa04659, FDR < 10^−15^), neutrophil degranulation (R-HSA-6798695, FDR < 10^−10^), and T cell selection (GO:0045058, FDR < 10^−7^), suggesting a larger involvement of adaptive immune responses in the respiratory infections related to bacterial pneumonia.

## Discussion

Using altogether nine independent transcriptomic datasets from diverse cohort studies and various types of blood samples, we discovered a signature of 149 genes consistently and specifically related to COVID-19, providing a comprehensive view of the specific disease-related host processes. The identified COVID-19 specific signature confirmed many processes previously associated with the disease in multiple studies, including induction of the immunoglobulin and hemostasis signals, as well as dysregulation of the cell cycle. Moreover, many specific genes previously associated with COVID-19 showed consistent dysregulation across multiple datasets, supporting their relevance in the disease.

In addition to genes previously associated with COVID-19, we also identified multiple genes that have not yet been widely studied in the context of COVID-19. These included, for instance, COVID-19 specific upregulation of tumor necrosis factor receptor superfamily members 13B and 17 (TNFRSF13B and TNFRSF17), predominantly found in B cells and involved in immune responses; upregulation of the regulator of G protein signaling 1 (RGS1), which has previously been linked to multiple immune-mediated diseases such as celiac disease, type 1 diabetes, and multiple sclerosis (Smyth et al., 2008; International Multiple Sclerosis Genetics Consortium et al., 2011); upregulation of the G protein-coupled receptor 84 (GPR84), which is a pro-inflammatory receptor that has previously been associated with inflammatory bowel disease (Planell et al., 2017); and downregulation of Fc receptor-like 2 (FCRL2), which encodes a member of the immunoglobulin receptor superfamily.

One of the strongest COVID-19 specific signals observed in this study was related to specific immunoglobulin genes, which were consistently upregulated in COVID-19 patients compared to healthy controls across multiple datasets. Furthermore, the discovered immunoglobulin genes were not similarly upregulated in other infections in the McClain data, suggesting a possible COVID-19 specific upregulation for the particular combination of the discovered immunoglobulin genes. Our findings were in agreement with those of McClain et al. (2021), who observed the immunoglobulin pathways and specific immunoglobulin-related genes as upregulated in COVID-19 patients when compared to other infections and healthy controls. Investigation of the immunoglobulin-related genes at the single-cell level suggested that the observed bulk signal came from B cells and plasmablasts (class-switched B cells), possibly reflecting a markedly strong induction of B cell differentiation to antibody-producing plasmablasts in COVID-19 patients. Immunoglobulin-related genes have been recently reported to be similarly upregulated in COVID-19 when compared to healthy patients and patients with active influenza infection (Bibert et al., 2021). The B cell-mediated humoral immune response plays a critical role in preventing and neutralizing COVID-19 infection and partly depends on the somatic recombination and differential usage of the immunoglobulin genes in producing a diverse repertoire of B cell receptors and associated antibodies (He et al., 2021). It is conceivable that a COVID-19 infection induces a strong immunoglobulin signal involving a distinct combination of immunoglobulin-related genes. However, even though suggestive, the extent to which the discovered immunoglobulin signal in this study is specific to COVID-19 and not other respiratory infections requires further confirmation from future studies.

The elevated expression of the immunoglobulin-related genes was observed in both severe and mild cases. While Overmyer et al. (2021) observed upregulation of many immunoglobulin genes in patients with severe COVID-19 when compared to those with mild disease, we did not observe systematic associations between immunoglobulin-related genes and disease severity across the datasets. The only exception was IGHV4-34, which was systematically upregulated in the severe cases in all three datasets, where it was detected. Curiously, IGHV4-34 has an inherent ability to encode autoreactive antibodies; IGHV4-34 antibodies represent a major proportion of serum antibodies, especially in systemic lupus erythematosus (SLE), and they are associated with the disease severity, while they are underrepresented in the serum of healthy adults (van Vollenhoven et al., 1999; Pugh-Bernard et al., 2001). In line with our study, increased usage of IGHV4-34 has also been observed in COVID-19 compared to healthy controls (Galson et al., 2020). It would be interesting to study whether autoreactivity contributes to the development of long COVID, with symptoms often resembling those observed in autoimmune diseases (Galson et al., 2020; Khamsi, 2021).

Immunoglobulin antibodies against the SARS-CoV-2 spike protein antigens have been shown to develop rapidly in individuals infected with the virus (Secchi et al., 2020). Intravenous immunoglobulin injections from healthy donors or recovering patients (Nabih, 2021) have been used to treat COVID-19 patients with promising results (Herth et al., 2020; Cao et al., 2021), with the rationale to suppress the hyperactive immune responses seen in patients with severe disease (Tzilas et al., 2020). Interestingly, PTX3 has been suggested as a biomarker for the unresponsiveness to intravenous immunoglobulin treatment of patients with Kawasaki disease, causing inflammation of blood vessels throughout the body (PTX3 and PREDICTS INTRAVENOUS IMMUNOGLOBULIN UNRESPONSIVENESS IN PATIENTS WITH KAWASAKI DISEASE, 2011; Kitoh et al., 2021). PTX3 is involved in humoral innate immunity and regulation of inflammation, including neutrophil recruitment and complement cascade regulation (Deban et al., 2008; Deban et al., 2010). Uncontrolled complement activation has been associated with severe COVID-19 (Risitano et al., 2020). Recently, PTX3 was identified as a predictor of 28-day mortality of hospitalized COVID-19 patients, with increased PTX3 hypothesized to reflect the failure to regulate uncontrolled inflammation (Brunetta et al., 2021).

Several previous studies have shown that the interferon response is elevated in COVID-19 when compared to healthy controls, but it appears to be less strongly induced in COVID-19 compared to other infections such as influenza (Lee et al., 2020; Wilk et al., 2020; Liu et al., 2021; McClain et al., 2021; Ng et al., 2021), especially in the severe disease (Combes et al., 2021; Liu et al., 2021). We did not observe an enrichment of genes directly related to the interferon response among our COVID-19 specific gene signature. Only one gene directly related to the interferon response was detected in our COVID-19 specific gene signature: interferon-alpha inducible protein 27 (IFI27). IFI27 was consistently up-regulated in COVID-19 when compared to the healthy controls, but the comparisons against other infections and in relation to disease severity varied depending on the dataset, perhaps reflecting inconsistent interferon response observed in previous studies. IFI27 was also strongly associated with the time from the symptom onset, with the initial high expression decreasing close to control levels relatively quickly.

Although the interferon signal among our COVID-19 specific genes was mostly absent, some upregulated chemokines were identified as COVID-19 specific. These included the C-X-C motif chemokine ligand 3 (CXCL3), which is involved in the migration and adhesion of monocytes (Smith et al., 2005), and the platelet factor 4 (PF4), which is also known as the C-X-C motif chemokine ligand 4 (CXCL4) and is a chemotaxis inducer for neutrophils, monocytes, and fibroblasts (Eisman et al., 1990). Chemokines have been suggested to be deeply involved in COVID-19 and even the main cause of the acute respiratory syndrome and cytokine storm associated with the most severe forms of the disease (Majumdar and Murphy, 2020; Coperchini et al., 2021; Khalil et al., 2021). Furthermore, neutrophilia (i.e., a high number of circulating neutrophils) (Coperchini et al., 2021; Ng et al., 2021), neutrophil degranulation (Ng et al., 2021; Overmyer et al., 2021), and high chemokine expression (Ng et al., 2021; Overmyer et al., 2021) have previously been associated with severe COVID-19.

Curiously, Wilk et al. (2020) observed a novel cell population of developing neutrophils in COVID-19. These neutrophils appeared similar to plasmablasts and neutrophil progenitors in their gene expression and were suspected to be possibly derived from plasmablasts or through emergency granulopoiesis (Wilk et al., 2020). Interestingly, LCN2 was identified to be upregulated in a COVID-19 specific manner in our analysis across the datasets and was preferentially expressed in that cell population. Recently, Meizlish et al. (2021) suggested LCN2 as a discriminator of critical illness in COVID-19, being highly enriched in neutrophil precursors in circulation. Earlier studies have implicated LCN2 to deactivate macrophages, worsening the inflammatory response and negatively affecting the outcome of pneumococcal pneumonia (Warszawska et al., 2013).

The identified COVID-19 specific signature involved a clear upregulation of hemostasis signal when compared to healthy controls. The signal was stronger in severe diseases, with many of the hemostasis-related genes consistently upregulated when comparing severe cases to milder diseases. This was well in line with previous studies. Several studies have shown significant increases in platelet activation, platelet reactivity, and platelet-leukocyte aggregates in COVID-19 compared to healthy blood donors (Hottz et al., 2020; Manne et al., 2020; Comer et al., 2021). Platelet activation has also been reported to correlate with COVID-19 severity (Hottz et al., 2020; Comer et al., 2021) and platelet hyperreactivity has been suggested as a primary driver of thrombosis contributing to organ failure and death in the severe disease (Zaid et al., 2021). Notably, a recent study reported that platelet activation was significantly higher in COVID-19 patients compared to patients with acute respiratory distress syndrome without COVID-19 (Zaid et al., 2021), supporting the COVID-19 specificity of the signal.

In association with hemostasis and platelet function, we also observed a highly enriched Reactome pathway “RUNX1 regulates genes involved in megakaryocyte differentiation and platelet function” (Matthews et al., 2009) in our COVID-19 specific signature. The related genes were consistently upregulated in COVID-19 patients when compared to healthy controls and other infections. RUNX1 is a transcription factor and a master regulator that is involved in the maturation of hematopoietic stem cells into mature cells, and it has been previously related to angiogenesis and fibrosis (O’Hare et al., 2021). Accumulated clonal mutations in hematopoietic stem cells have been associated with an increased risk of severe COVID-19 (Bolton et al., 2020). Moreover, inhibition of RUNX1 has been shown to enhance symptoms of lung fibrosis in a mouse model (O’Hare et al., 2021), while overexpression of RUNX1 has been observed in the lungs of severe COVID-19 patients who died of the disease, with widespread thrombosis and microangiopathy and related vascular angiogenesis much more prevalent in COVID-19 than in influenza (Ackermann et al., 2020). In line with this, the RUNX1-related genes of our COVID-19 signature were mostly detected as upregulated in the severe form of the disease when compared to the milder disease.

Neutrophils are known to interact extensively with platelets during inflammatory conditions, and they modulate each other’s functions (Lisman, 2018; Ramirez et al., 2019; Zucoloto and Jenne, 2019). Among such interactions, platelets have been shown to induce the formation of neutrophil extracellular traps, which are known to kill pathogens but also be involved in thrombin activation and coagulation initiation, which has been associated with hypercoagulability in vascular disorders (Zucoloto and Jenne, 2019). Interestingly, COVID-19 has been strongly associated with a hypercoagulative phenotype (Becker, 2020; Goshua et al., 2020), and thrombosis (Ackermann et al., 2020; McClain et al., 2021), and widespread microangiopathy in the lungs (Ackermann et al., 2020). Furthermore, while platelets are well known to be associated with hemostasis (Lisman, 2018; Zucoloto and Jenne, 2019), the myriad of interactions between the two cell types also suggests the involvement of neutrophils in hemostasis and blood coagulation during inflammatory conditions (Lisman, 2018; Ramirez et al., 2019; Zucoloto and Jenne, 2019).

Another clear signal detected among our COVID-19 specific genes was related to cell cycle and mitotic division control. Coronaviruses in general have been shown to manipulate the cell cycle of the host cells, especially the arrest of the cell cycle at specific cell cycle checkpoints (Dove et al., 2006; Simabuco et al., 2020; Su et al., 2020) and also mitotic events in COVID-19 (Bock and Ortea, 2020). Furthermore, several mRNA molecules related to the cell cycle and mitotic processes have been observed to be upregulated in response to COVID-19 infection (Bouhaddou et al., 2020). For instance, CDKN1A has been observed to have significantly higher expression in COVID-19 patients than in healthy controls (Bordoni et al., 2021), which is in agreement with our results of COVID-19 specific CDKN1A upregulation. The protein product of CDKN1A, p21, is an essential mediator of p53-dependent cell arrest (Bordoni et al., 2021).

While multiple genes in our COVID-19 specific signature were associated with disease severity, their association with time from symptom onset was less evident, as could be expected. Among the genes showing association, the expression changes typically tended to become closer to the healthy controls over time, with two outstanding exceptions: TRDC and PPIF. TRDC encodes the constant region of the T cell receptor delta chain, and it is considered a marker of gamma-delta T cells. In line with our finding that the expression of TRDC decreased in the blood of COVID-19 patients, previous studies have suggested decreased proportions of gamma-delta T cells in the blood of hospitalized COVID-19 patients compared to healthy controls (Wilk et al., 2020). The decrease has been associated with their recruitment to airway tissues (Caron et al., 2021) and disease severity (Zhang et al., 2020b).

PPIF, whose expression increased in the blood of COVID-19 patients, is a cyclophilin that is an essential component of the mitochondrial permeability transition pore. The opening of the pore has been implicated in the pathophysiology of multiple diseases, such as muscular dystrophies, ischemia-reperfusion injury, and various neurological diseases, while inhibition of PPIF has been suggested as a therapeutic strategy to delay it (Briston et al., 2019). Intriguingly, one of our identified top drug candidates was cyclosporin A, which is indeed a cyclophilin inhibitor. Cyclosporin A is widely used to prevent organ rejection after transplantation, but it has recently been shown to have substantial antiviral activity against SARS-CoV-2 and preliminary clinical trials on COVID-19 patients have reported a lower incidence of death among the cyclosporin A treated patients, recently reviewed by (Devaux et al., 2021).

Overall, an investigation of the associations of the COVID-19 specific transcriptomic signature with known drug and chemical compound signatures identified several drugs and chemical compounds with known relations to COVID-19, providing further support for the relevance of our COVID-19 specific signature. Additionally, this provided opportunities to gain further insights for possible new drug relationships with the disease, while further investigations of the findings are needed to provide the rationale for their potential in COVID-19 treatment.

In addition to the newly discovered COVID-19 specific gene signature, we explored genes similarly regulated in COVID-19 and other viral or bacterial infections when compared to healthy controls. The majority of these genes were down-regulated and they were highly enriched in ribosome-related processes. Congruently to our results, different viruses such as influenza (Bercovich-Kinori et al., 2016), HIV-1 (Kleinman et al., 2014), vaccinia (Dai et al., 2017), and SARS-CoV2 (Banerjee et al., 2020; Hsu et al., 2021) have been observed to be related to a global inhibition of the host mRNA translation upon infection and, as such, might represent a common strategy employed by several viruses to shut down the native host protein synthesis (Bercovich-Kinori et al., 2016; Hsu et al., 2021). Similarly, the bacterial agent *Legionella pneumophila*, causative of pneumonia in humans, has been observed to target and inhibit host mRNA translation and protein synthesis (Belyi, 2020).

Taken together, our results offer a rich resource to comprehensively investigate the COVID-19 specific host responses in circulating blood, providing support for many signals previously associated with the disease and a solid foundation for future research into the specific mechanisms related to COVID-19. To facilitate such exploration, we also offer a web-based software platform enabling information-rich visualization of the transcriptomic profiles across multiple datasets at both bulk and single-cell levels. The expression of specific genes can be compared between COVID-19 patients and healthy controls, as well as patients with other infections. Gene expression can also be investigated in relation to many relevant attributes, such as age, sex, disease severity, disease progression, and cell-type specificity. The software platform is freely available at https://elolab.shinyapps.io/COVID19/.

## Methods

### Transcriptomic datasets and their preprocessing

The transcriptomic datasets used in this study (Table 1) were downloaded from the Gene Expression Omnibus (GEO) as raw count matrices, except for the monocyte Brunetta data, for which only the preprocessed data was available, and the single-cell Wilk data, which was downloaded from the COVID-19 cell Atlas (https://www.covid19cellatlas.org) as preprocessed data.

The single-cell Lee and Combes datasets were processed using Seurat (v.4.0.1) in R similarly to in the original publications (Lee et al., 2020; Combes et al., 2021). Cell type annotations for all the single-cell datasets were performed using the Azimuth tool with the human PBMC reference (Hao et al., 2021). Since the PBMC reference did not include neutrophils, the neutrophil annotations for the whole-blood Combes data were retrieved from the original study. For the bulk analysis, the raw gene-wise count values from all cells belonging to a sample were aggregated using the R package Muscat (v.1.4.0), resulting in a pseudobulk expression matrix with genes as rows and samples as columns. To identify gene signals comparable to those from bulk RNA-seq datasets, the raw count values were aggregated across all cells belonging to one sample (Crowell et al., 2020).

All bulk and pseudobulk RNA-seq datasets were preprocessed as similarly as possible from the raw count matrices. First, lowly expressed genes were filtered out, retaining only genes that had a count per million (CPM) value above the threshold in at least as many samples as the size of the smallest experimental group in the data. The threshold was determined for each dataset as the CPM value corresponding to the read count of ten in the sample with the smallest library size. The data were normalized using the trimmed mean of M-values (TMM) method from the Bioconductor package edgeR (v.3.26.8). For the analysis, we used log_2_ transformed CPM values with an offset of 1.

In the Lee data, one influenza sample (“Flu 5”) was removed as an outlier. In the Combes data, nine samples (one healthy control, three COVID-19 positives, and five COVID-19 negatives) were excluded because they contained less than 1,000 cells.

### Defining the COVID-19-specific gene signature

For defining the COVID-19 specific gene signature, we used the whole blood McClain data (McClain et al., 2021) and the PBMC Lee data (Lee et al., 2020). Only one sample per individual was considered in the analysis; if an individual had multiple samples, their median was used for each gene. The reproducibility optimized test statistic (ROTS) (v.1.12.0) (Suomi et al., 2017) was first applied to determine differentially expressed genes between the COVID-19 cases and healthy controls separately in both datasets. Genes with a false discovery rate (FDR) of 0.05 were considered differentially expressed. To focus on COVID-19 specific signals, we then identified those genes that were differentially expressed between COVID-19 patients and healthy controls but not similarly differentially expressed in the same direction between any other disease state (influenza, seasonal coronavirus, and bacterial pneumonia) and healthy controls. Finally, the union of these COVID-19 specific genes was further refined using six additional datasets by including only those genes that had a concordant fold change in at least four of the datasets and an opposite change in at most one dataset in our final COVID-19 specific signature.

### Association of the COVID-19-specific gene signature with disease severity and time from symptom onset

For the exploration of disease severity, we used those six datasets (McClain, Lee, Arunachalam, Combes, Liu, Overmeyer) that had severity information available. The patients were divided into two categories based on whether they required mechanical ventilation or intensive care unit care (severe) or not (mild) according to the original studies. All the patients in both categories were hospitalized apart from patients in the mild category in the McClain data. To determine the significance of differences between the severe and mild cases, Wilcoxon rank-sum test was used. For functional enrichment analysis, genes consistently upregulated in severe disease in at least four datasets were considered.

For determining the association of the COVID-19 specific genes with time from symptom onset, we considered those four (Arunachalam, Combes, Liu, Wilk) datasets that had the symptom onset information available. The associations were determined using Pearson correlation between the reported number of days from the symptom onset and the measured gene expression level, scaled by the corresponding control average.

### Functional enrichment and associations with known drugs and chemical perturbations

To explore functional enrichment in the detected COVID-19 specific gene signature, we used the Metascape platform (Zhou et al., 2019). The following ontology sources were considered: KEGG Pathways, GO Biological Processes, Reactome Gene Sets, Canonical Pathways, and WikiPathways. All genes in the genome were used as the background. Metascape calculates the statistical significance of enrichment using the hypergeometric distribution and adjusts for multiple testing using the Benjamini-Hochberg method. Terms with a *p*-value < 0.01, a minimum of three genes, and an enrichment of at least 1.5 are further grouped into clusters using hierarchical clustering with Kappa scores as the similarity measure. Sub-trees with a similarity of >0.3 were considered a cluster, and the most significant term within a cluster was used to represent the cluster.

For investigating the associations of the COVID-19 specific signature genes with known drugs and chemical compound signatures, we used the Library of Integrated Network-based Cellular Signatures (LINCS) database and the associated integrative web-based platform (iLINCS) (Koleti et al., 2017; Keenan et al., 2018). The upregulated and downregulated COVID-19 specific genes were used as the query signatures and the DrugMatrix and pharmacogenomics transcriptional signatures as the iLINCS signatures. Concordance was determined on the basis of the correlation between the query signature and the iLINCS signatures. Signatures with *p* < 0.01 and a negative concordance value below −0.35 were considered.

### Cell type proportions and cell type-specific expression

The Wilcoxon rank-sum test was used to calculate the statistical significance of differences in the cell type proportions between the COVID-19 patients and healthy controls in the single-cell datasets. To investigate which cell types contributed most to the observed bulk expression of the signature genes, the sequencing reads were assigned to different cell types and their relative proportions across the cell types were calculated.

## Data Availability

Publicly available datasets were analyzed in this study. These data can be found at: GSE161731, GSE149689, GSE163151, GSE163668, GSE157103, GSE152418, GSE161918, GSE150728, and GSE160351.

## References

[B1] Ackermann M. Verleden S. E. Kuehnel M. Haverich A. Welte T. Laenger F. (2020). Pulmonary vascular endothelialitis, thrombosis, and angiogenesis in covid-19. N. Engl. J. Med. 383, 120–128. 10.1056/NEJMoa2015432 32437596 PMC7412750

[B2] Ahmadi A. Moradi S. (2021). *In silico* analysis suggests the RNAi-enhancing antibiotic enoxacin as a potential inhibitor of SARS-CoV-2 infection. Sci. Rep. 11, 10271. 10.1038/s41598-021-89605-6 33986351 PMC8119475

[B3] Ahmed M. Z. Zia Q. Haque A. Alqahtani A. S. Almarfadi O. M. Banawas S. (2021). Aminoglycosides as potential inhibitors of SARS-CoV-2 main protease: An *in silico* drug repurposing study on FDA-approved antiviral and anti-infection agents. J. Infect. Public Health 14, 611–619. 10.1016/j.jiph.2021.01.016 33866129 PMC7871101

[B4] Arunachalam P. S. Wimmers F. Mok C. K. P. Perera R. A. P. M. Scott M. Hagan T. (2020). Systems biological assessment of immunity to mild versus severe COVID-19 infection in humans. Science 369, 1210–1220. 10.1126/science.abc6261 32788292 PMC7665312

[B5] Banerjee A. K. Blanco M. R. Bruce E. A. Honson D. D. Chen L. M. Chow A. (2020). SARS-CoV-2 disrupts splicing, translation, and protein trafficking to suppress host defenses. Cell. 183, 1325–1339. e21. 10.1016/j.cell.2020.10.004 33080218 PMC7543886

[B6] Becker R. C. (2020). COVID-19 update: Covid-19-Associated coagulopathy. J. Thromb. Thrombolysis 50, 54–67. 10.1007/s11239-020-02134-3 32415579 PMC7225095

[B7] Belyi Y. (2020). Targeting eukaryotic mRNA translation by *Legionella pneumophila* . Front. Mol. Biosci. 7, 80. 10.3389/fmolb.2020.00080 32411722 PMC7201127

[B8] Bercovich-Kinori A. Tai J. Gelbart I. A. Shitrit A. Ben-Moshe S. Drori Y. (2016). A systematic view on influenza induced host shutoff. Elife 5, e18311. 10.7554/eLife.18311 27525483 PMC5028189

[B9] Bibert S. Guex N. Lourenco J. Brahier T. Papadimitriou-Olivgeris M. Damonti L. (2021). Transcriptomic signature differences between SARS-CoV-2 and influenza virus infected patients. Front. Immunol. 0, 666163. 10.3389/fimmu.2021.666163 PMC8202013 34135895

[B10] Bock J-O. Ortea I. (2020). Re-Analysis of SARS-CoV-2-infected host cell proteomics time-course data by impact pathway analysis and network analysis: A potential link with inflammatory response. Aging 12, 11277–11286. 10.18632/aging.103524 32575076 PMC7343490

[B11] Bolton K. L. Koh Y. Foote M. B. Im H. Jee J. Sun C. H. (2020). Clonal hematopoiesis is associated with risk of severe Covid-19. medRxiv., 2020.11.25.20233163. 10.1101/2020.11.25.20233163 PMC8514469 34645798

[B12] Bordoni V. Tartaglia E. Sacchi A. Fimia G. M. Cimini E. Casetti R. (2021). The unbalanced p53/SIRT1 axis may impact lymphocyte homeostasis in COVID-19 patients. Int. J. Infect. Dis. 105, 49–53. 10.1016/j.ijid.2021.02.019 33578018 PMC7872850

[B13] Bouhaddou M. Memon D. Meyer B. White K. M. Rezelj V. V. Correa Marrero M. (2020). The global phosphorylation landscape of SARS-CoV-2 infection. Cell. 182, 685–712. 10.1016/j.cell.2020.06.034 32645325 PMC7321036

[B14] Briston T. Selwood D. L. Szabadkai G. Duchen M. R. (2019). Mitochondrial permeability transition: A molecular lesion with multiple drug targets. Trends Pharmacol. Sci. 40, 50–70. 10.1016/j.tips.2018.11.004 30527591

[B15] Brunetta E. Folci M. Bottazzi B. De Santis M. Gritti G. Protti A. (2021). Macrophage expression and prognostic significance of the long pentraxin PTX3 in COVID-19. Nat. Immunol. 22, 19–24. 10.1038/s41590-020-00832-x 33208929

[B16] Cao W. Liu X. Hong K. Ma Z. Zhang Y. Lin L. (2021). High-dose intravenous immunoglobulin in severe coronavirus disease 2019: A multicenter retrospective study in China. Front. Immunol. 12, 627844. 10.3389/fimmu.2021.627844 33679771 PMC7933558

[B17] Caron J. Ridgley L. A. Bodman-Smith M. (2021). How to train your dragon: Harnessing gamma delta T cells antiviral functions and trained immunity in a pandemic era. Front. Immunol. 12, 666983. 10.3389/fimmu.2021.666983 33854516 PMC8039298

[B18] Choi E. S. Nichol J. L. Hokom M. M. Hornkohl A. C. Hunt P. (1995). Platelets generated *in vitro* from proplatelet-displaying human megakaryocytes are functional. Blood 85, 402–413. 10.1182/blood.V85.2.402.402 7529062

[B19] Combes A. J. Courau T. Kuhn N. F. Hu K. H. Ray A. Chen W. S. (2021). Global absence and targeting of protective immune states in severe COVID-19. Nature 591, 124–130. 10.1038/s41586-021-03234-7 33494096 PMC8567458

[B20] Comer S. P. Cullivan S. Szklanna P. B. Weiss L. Cullen S. Kelliher S. (2021). COVID-19 induces a hyperactive phenotype in circulating platelets. PLoS Biol. 19, e3001109. 10.1371/journal.pbio.3001109 33596198 PMC7920383

[B21] Coperchini F. Chiovato L. Ricci G. Croce L. Magri F. Rotondi M. (2021). The cytokine storm in COVID-19: Further advances in our understanding the role of specific chemokines involved. Cytokine Growth Factor Rev. 58, 82–91. 10.1016/j.cytogfr.2020.12.005 33573850 PMC7837329

[B22] Crowell H. L. Soneson C. Germain P-L. Calini D. Collin L. Raposo C. (2020). Muscat detects subpopulation-specific state transitions from multi-sample multi-condition single-cell transcriptomics data. Nat. Commun. 11, 6077. 10.1038/s41467-020-19894-4 33257685 PMC7705760

[B23] Dai A. Cao S. Dhungel P. Luan Y. Liu Y. Xie Z. (2017). Ribosome profiling reveals translational upregulation of cellular oxidative phosphorylation mRNAs during vaccinia virus-induced host shutoff. J. Virol. 91, e01858-16. 10.1128/JVI.01858-16 28003488 PMC5309933

[B24] Dalhoff A. (2015). Antiviral, antifungal, and antiparasitic activities of fluoroquinolones optimized for treatment of bacterial infections: A puzzling paradox or a logical consequence of their mode of action? Eur. J. Clin. Microbiol. Infect. Dis. 34, 661–668. 10.1007/s10096-014-2296-3 25515946 PMC7087824

[B25] Deban L. Jarva H. Lehtinen M. J. Bottazzi B. Bastone A. Doni A. (2008). Binding of the long pentraxin PTX3 to factor H: Interacting domains and function in the regulation of complement activation. J. Immunol. 181, 8433–8440. 10.4049/jimmunol.181.12.8433 19050261

[B26] Deban L. Russo R. C. Sironi M. Moalli F. Scanziani M. Zambelli V. (2010). Regulation of leukocyte recruitment by the long pentraxin PTX3. Nat. Immunol. 11, 328–334. 10.1038/ni.1854 20208538

[B27] Devaux C. A. Melenotte C. Piercecchi-Marti M-D. Delteil C. Raoult D. (2021). Cyclosporin A: A repurposable drug in the treatment of COVID-19? Front. Med. 8, 663708. 10.3389/fmed.2021.663708 PMC8450353 34552938

[B28] Dove B. Brooks G. Bicknell K. Wurm T. Hiscox J. A. (2006). Cell cycle perturbations induced by infection with the coronavirus infectious bronchitis virus and their effect on virus replication. J. Virol. 80, 4147–4156. 10.1128/JVI.80.8.4147-4156.2006 16571830 PMC1440480

[B29] Eisman R. Surrey S. Ramachandran B. Schwartz E. Poncz M. (1990). Structural and functional comparison of the genes for human platelet factor 4 and PF4alt. Blood 76, 336–344. Available: https://www.ncbi.nlm.nih.gov/pubmed/1695112 . 10.1182/blood.v76.2.336.bloodjournal762336 1695112

[B30] Elbadwi F. A. Khairy E. A. Alsamani F. O. Mahadi M. A. Abdalrahman S. E. Ahmed Z. A. M. (2021). Identification of novel transmembrane Protease Serine Type 2 drug candidates for COVID-19 using computational studies. Inf. Med. Unlocked 26, 100725. 10.1016/j.imu.2021.100725 PMC8421083 34514079

[B31] Galson J. D. Schaetzle S. Bashford-Rogers R. J. M. Raybould M. I. J. Kovaltsuk A. Kilpatrick G. J. (2020). Deep sequencing of B cell receptor repertoires from COVID-19 patients reveals strong convergent immune signatures. Front. Immunol. 11, 605170. 10.3389/fimmu.2020.605170 33384691 PMC7769841

[B32] Goshua G. Pine A. B. Meizlish M. L. Chang C-H. Zhang H. Bahel P. (2020). Endotheliopathy in COVID-19-associated coagulopathy: Evidence from a single-centre, cross-sectional study. Lancet. Haematol. 7, e575–e582. 10.1016/S2352-3026(20)30216-7 32619411 PMC7326446

[B33] Guan W-J. Ni Z-Y. Hu Y. Liang W-H. Ou C-Q. He J-X. (2020). Clinical characteristics of coronavirus disease 2019 in China. N. Engl. J. Med. 382, 1708–1720. 10.1056/nejmoa2002032 32109013 PMC7092819

[B34] Guisado-Vasco P. Valderas-Ortega S. Carralón-González M. M. Roda-Santacruz A. González-Cortijo L. Sotres-Fernández G. (2020). Clinical characteristics and outcomes among hospitalized adults with severe COVID-19 admitted to a tertiary medical center and receiving antiviral, antimalarials, glucocorticoids, or immunomodulation with tocilizumab or cyclosporine: A retrospective observational study (coquima cohort). EClinicalMedicine 28, 100591. 10.1016/j.eclinm.2020.100591 33078138 PMC7557296

[B35] Hao Y. Hao S. Andersen-Nissen E. Mauck W. M. 3rd Zheng S. Butler A. (2021). Integrated analysis of multimodal single-cell data. Cell. 184, 3573–3587.e29. e29. 10.1016/j.cell.2021.04.048 34062119 PMC8238499

[B36] He B. Liu S. Wang Y. Xu M. Cai W. Liu J. (2021). Rapid isolation and immune profiling of SARS-CoV-2 specific memory B cell in convalescent COVID-19 patients via LIBRA-seq. Signal Transduct. Target. Ther. 6, 195. 10.1038/s41392-021-00610-7 34001847 PMC8127497

[B37] Herth F. J. F. Sakoulas G. Haddad F. (2020). Use of intravenous immunoglobulin (prevagen or octagam) for the treatment of COVID-19: Retrospective case series. Respiration. 99, 1145–1153. 10.1159/000511376 33316806 PMC7801971

[B38] Hippisley-Cox J. Young D. Coupland C. Channon K. M. Tan P. S. Harrison D. A. (2020). Risk of severe COVID-19 disease with ACE inhibitors and angiotensin receptor blockers: Cohort study including 8.3 million people. Heart 106, 1503–1511. 10.1136/heartjnl-2020-317393 32737124 PMC7509391

[B39] Hottz E. D. Azevedo-Quintanilha I. G. Palhinha L. Teixeira L. Barreto E. A. Pão C. R. R. (2020). Platelet activation and platelet-monocyte aggregate formation trigger tissue factor expression in patients with severe COVID-19. Blood 136, 1330–1341. 10.1182/blood.2020007252 32678428 PMC7483437

[B40] Hsu J. C-C. Laurent-Rolle M. Pawlak J. B. Wilen C. B. Cresswell P. (2021). Translational shutdown and evasion of the innate immune response by SARS-CoV-2 NSP14 protein. Proc. Natl. Acad. Sci. U. S. A., e2101161118. 10.1073/pnas.2101161118 34045361 PMC8214666

[B41] Huang I. Pranata R. (2020). Lymphopenia in severe coronavirus disease-2019 (COVID-19): Systematic review and meta-analysis. J. Intensive Care 8, 36. 10.1186/s40560-020-00453-4 32483488 PMC7245646

[B42] Ikeda S. Yazawa M. Nishimura C. (1987). Antiviral activity and inhibition of topoisomerase by ofloxacin, a new quinolone derivative. Antivir. Res. 8, 103–113. 10.1016/0166-3542(87)90064-7 2827566

[B43] Jang Y. Shin J. S. Lee M. K. Jung E. An T. Kim U-I. (2021). Comparison of antiviral activity of gemcitabine with 2’-fluoro-2'-deoxycytidine and combination therapy with remdesivir against SARS-CoV-2. Int. J. Mol. Sci. 22, 1581. 10.3390/ijms22041581 33557278 PMC7915419

[B44] Karampela I. Dalamaga M. (2020). Could respiratory fluoroquinolones, levofloxacin and moxifloxacin, prove to be beneficial as an adjunct treatment in COVID-19? Arch. Med. Res. 51, 741–742. 10.1016/j.arcmed.2020.06.004 32546446 PMC7275144

[B45] Keenan A. B. Jenkins S. L. Jagodnik K. M. Koplev S. He E. Torre D. (2018). The library of integrated network-based cellular signatures NIH program: System-level cataloging of human cells response to perturbations. Cell. Syst. 6, 13–24. 10.1016/j.cels.2017.11.001 29199020 PMC5799026

[B46] Keith P. Saint-Jour M. Pusey F. Hodges J. Jalali F. Scott L. K. (2021). Unprovoked serotonin syndrome-like presentation of SARS-CoV-2 infection: A small case series. SAGE Open Med. Case Rep. 9, 2050313X211032089. 10.1177/2050313X211032089 PMC8274092 34290872

[B47] Khalil B. A. Elemam N. M. Maghazachi A. A. (2021). Chemokines and chemokine receptors during COVID-19 infection. Comput. Struct. Biotechnol. J. 19, 976–988. 10.1016/j.csbj.2021.01.034 33558827 PMC7859556

[B48] Khamsi R. (2021). Rogue antibodies could be driving severe COVID-19. Nature 590, 29–31. 10.1038/d41586-021-00149-1 33469204

[B49] Kitoh T. Ohara T. Muto T. Okumura A. Baba R. Koizumi Y. (2021). Increased pentraxin 3 levels correlate with IVIG responsiveness and coronary artery aneurysm formation in Kawasaki disease. Front. Immunol. 12, 624802. 10.3389/fimmu.2021.624802 33912155 PMC8072470

[B50] Kleinman C. L. Doria M. Orecchini E. Giuliani E. Galardi S. De Jay N. (2014). HIV-1 infection causes a down-regulation of genes involved in ribosome biogenesis. PLoS One 9, e113908. 10.1371/journal.pone.0113908 25462981 PMC4252078

[B51] Koleti A. Terryn R. Stathias V. Chung C. Cooper D. J. Turner J. P. (2017). Data portal for the library of integrated network-based cellular signatures (LINCS) program: Integrated access to diverse large-scale cellular perturbation response data. Nucleic Acids Res. 46, D558-D566. 10.1093/nar/gkx1063 PMC5753343 29140462

[B52] Lee J. S. Park S. Jeong H. W. Ahn J. Y. Choi S. J. Lee H. (2020). Immunophenotyping of COVID-19 and influenza highlights the role of type I interferons in development of severe COVID-19. Sci. Immunol. 5, eabd1554. 10.1126/sciimmunol.abd1554 32651212 PMC7402635

[B53] Lisman T. (2018). Platelet-neutrophil interactions as drivers of inflammatory and thrombotic disease. Cell. Tissue Res. 371, 567–576. 10.1007/s00441-017-2727-4 29178039 PMC5820397

[B54] Liu C. Martins A. J. Lau W. W. Rachmaninoff N. Chen J. Imberti L. (2021). Time-resolved systems immunology reveals a late juncture linked to fatal COVID-19. Cell. 184, 1836–1857.e22. e22. 10.1016/j.cell.2021.02.018 33713619 PMC7874909

[B55] Majumdar S. Murphy P. M. (2020). Chemokine regulation during epidemic coronavirus infection. Front. Pharmacol. 11, 600369. 10.3389/fphar.2020.600369 33613280 PMC7890195

[B56] Manne B. K. Denorme F. Middleton E. A. Portier I. Rowley J. W. Stubben C. (2020). Platelet gene expression and function in patients with COVID-19. Blood 136, 1317–1329. 10.1182/blood.2020007214 32573711 PMC7483430

[B57] Marciniec K. Beberok A. Pęcak P. Boryczka S. Wrześniok D. (2020). Ciprofloxacin and moxifloxacin could interact with SARS-CoV-2 protease: Preliminary *in silico* analysis. Pharmacol. Rep. 72, 1553–1561. 10.1007/s43440-020-00169-0 33063271 PMC7561236

[B58] Matthews L. Gopinath G. Gillespie M. Caudy M. Croft D. de Bono B. (2009). Reactome knowledgebase of human biological pathways and processes. Nucleic Acids Res. 37, D619–D622. 10.1093/nar/gkn863 18981052 PMC2686536

[B59] McClain M. T. Constantine F. J. Henao R. Liu Y. Tsalik E. L. Burke T. W. (2021). Dysregulated transcriptional responses to SARS-CoV-2 in the periphery. Nat. Commun. 12, 1079. 10.1038/s41467-021-21289-y 33597532 PMC7889643

[B60] Meizlish M. L. Pine A. B. Bishai J. D. Goshua G. Nadelmann E. R. Simonov M. (2021). A neutrophil activation signature predicts critical illness and mortality in COVID-19. Blood Adv. 5, 1164–1177. 10.1182/bloodadvances.2020003568 33635335 PMC7908851

[B61] Metlay J. P. Waterer G. W. (2020). Treatment of community-acquired pneumonia during the coronavirus disease 2019 (COVID-19) pandemic. Ann. Intern. Med. 173, 304–305. 10.7326/M20-2189 32379883 PMC7236892

[B62] Montaño L. M. Sommer B. Gomez-Verjan J. C. Morales-Paoli G. S. Ramírez-Salinas G. L. Solís-Chagoyán H. (2022). Theophylline: Old drug in a new light, application in COVID-19 through computational studies. Int. J. Mol. Sci. 23, 4167. 10.3390/ijms23084167 35456985 PMC9030606

[B63] Nabih H. K. (2021). Importance of immunoglobulin therapy for COVID-19 patients with lymphocytopenia. Bull. Natl. Res. Cent. 45, 46. 10.1186/s42269-021-00502-4 33642851 PMC7897881

[B64] Nahmias Y. Ehrlich A. Ioannidis K. Nasar M. Alkian I. A. Hofree M. (2021). Metabolic regulation of SARS-CoV-2 infection. In Research square. Durham, NC, United States: Research Square. 10.21203/rs.3.rs-770724/v1

[B65] Ng D. L. Granados A. C. Santos Y. A. Servellita V. Goldgof G. M. Meydan C. (2021). A diagnostic host response biosignature for COVID-19 from RNA profiling of nasal swabs and blood. Sci. Adv. 7, eabe5984. 10.1126/sciadv.abe5984 33536218 PMC7857687

[B66] O’Hare M. Amarnani D. Whitmore H. A. B. An M. Marino C. Ramos L. (2021). Targeting runt-related transcription factor 1 prevents pulmonary fibrosis and reduces expression of severe acute respiratory syndrome coronavirus 2 host mediators. Am. J. Pathol. 191, 1193–1208. 10.1016/j.ajpath.2021.04.006 33894177 PMC8059259

[B67] Okuda T. Nishimura M. Nakao M. Fujita Y. (2001). RUNX1/AML1: A central player in hematopoiesis. Int. J. Hematol. 74, 252–257. 10.1007/BF02982057 11721959

[B68] Ong E. Z. Chan Y. F. Z. Leong W. Y. Lee N. M. Y. Kalimuddin S. Haja Mohideen S. M. (2020). A dynamic immune response shapes COVID-19 progression. Cell. Host Microbe 27, 879–882. e2. 10.1016/j.chom.2020.03.021 32359396 PMC7192089

[B69] Overmyer K. A. Shishkova E. Miller I. J. Balnis J. Bernstein M. N. Peters-Clarke T. M. (2021). Large-Scale multi-omic analysis of COVID-19 severity. Cell. Syst. 12, 23–40.e7. e7. 10.1016/j.cels.2020.10.003 33096026 PMC7543711

[B70] Pedersen S. F. Ho Y-C. (2020). SARS-CoV-2: A storm is raging. J. Clin. Investig. 130, 2202–2205. 10.1172/JCI137647 32217834 PMC7190904

[B71] Planell N. Masamunt M. C. Leal R. F. Rodríguez L. Esteller M. Lozano J. J. (2017). Usefulness of transcriptional blood biomarkers as a non-invasive surrogate marker of mucosal healing and endoscopic response in ulcerative colitis. J. Crohns Colitis 11, 1335–1346. 10.1093/ecco-jcc/jjx091 28981629 PMC5881703

[B72] Prajapat M. Shekhar N. Sarma P. Avti P. Singh S. Kaur H. (2020). Virtual screening and molecular dynamics study of approved drugs as inhibitors of spike protein S1 domain and ACE2 interaction in SARS-CoV-2. J. Mol. Graph. Model. 101, 107716. 10.1016/j.jmgm.2020.107716 32866780 PMC7442136

[B73] PREDICTS INTRAVENOUS IMMUNOGLOBULIN UNRESPONSIVENESS IN PATIENTS WITH KAWASAKI DISEASE (2011). Predicts intravenous immunoglobulin unresponsiveness in patients with Kawasaki disease. J. Am. Coll. Cardiol. 57, E2038. 10.1016/S0735-1097(11)62038-X 16735679

[B74] Pugh-Bernard A. E. Silverman G. J. Cappione A. J. Villano M. E. Ryan D. H. Insel R. A. (2001). Regulation of inherently autoreactive VH4-34 B cells in the maintenance of human B cell tolerance. J. Clin. Investig. 108, 1061–1070. 10.1172/JCI12462 11581307 PMC200949

[B75] Ramirez G. A. Manfredi A. A. Maugeri N. (2019). Misunderstandings between platelets and neutrophils build in chronic inflammation. Front. Immunol. 10, 2491. 10.3389/fimmu.2019.02491 31695699 PMC6817594

[B76] Raymonda M. H. Ciesla J. H. Monaghan M. Leach J. Asantewaa G. Smorodintsev-Schiller L. A. (2020). Pharmacologic profiling reveals lapatinib as a novel antiviral against SARS-CoV-2 *in vitro* . Biorxiv. 10.1101/2020.11.25.398859 PMC8626825 34871905

[B77] Resource Coordinators N. C. B. I. Agarwala R. Barrett T. Beck J. Benson D. A. Bollin C. (2017). Database resources of the national center for biotechnology information. Nucleic Acids Res. 46, D8-D13. 10.1093/nar/gkx1095 PMC5753372 29140470

[B78] Risitano A. M. Mastellos D. C. Huber-Lang M. Yancopoulou D. Garlanda C. Ciceri F. (2020). Complement as a target in COVID-19? Nat. Rev. Immunol. 20, 343–344. 10.1038/s41577-020-0320-7 32327719 PMC7187144

[B79] Russell T. W. Hellewell J. Jarvis C. I. van Zandvoort K. Abbott S. Ratnayake R. (20202020). Estimating the infection and case fatality ratio for coronavirus disease (COVID-19) using age-adjusted data from the outbreak on the Diamond Princess cruise ship, February 2020. Euro Surveill., 25. 10.2807/1560-7917.ES.2020.25.12.2000256 PMC7118348 32234121

[B80] Sauerhering L. Kuznetsova I. Kupke A. Meier L. Halwe S. Rohde C. (2022). Cyclosporin A reveals potent antiviral effects in preclinical models of SARS-CoV-2 infection. Am. J. Respir. Crit. Care Med. 205, 964–968. 10.1164/rccm.202108-1830LE 35167409 PMC9838622

[B81] International Multiple Sclerosis Genetics Consortium, Wellcome Trust Case Control Consortium 2 Sawcer S. Hellenthal G. Pirinen M. Spencer C. C. A. Patsopoulos N. A. Moutsianas L. (2011). Genetic risk and a primary role for cell-mediated immune mechanisms in multiple sclerosis. Nature 476, 214–219. 10.1038/nature10251 21833088 PMC3182531

[B82] Scroggs S. L. P. Andrade C. C. Chinnasamy R. Azar S. R. Schirtzinger E. E. Garcia E. I. (2020). Old drugs with new tricks: Efficacy of fluoroquinolones to suppress replication of flaviviruses. Viruses 12, E1022. 10.3390/v12091022 32933138 PMC7551155

[B83] Secchi M. Bazzigaluppi E. Brigatti C. Marzinotto I. Tresoldi C. Rovere-Querini P. (2020). COVID-19 survival associates with the immunoglobulin response to the SARS-CoV-2 spike receptor binding domain. J. Clin. Investig. 130, 6366–6378. 10.1172/JCI142804 32991329 PMC7685720

[B84] Seyednasrollah F. Rantanen K. Jaakkola P. Elo L. L. (2016). Rots: Reproducible RNA-seq biomarker detector-prognostic markers for clear cell renal cell cancer. Nucleic Acids Res. 44, e1. 10.1093/nar/gkv806 26264667 PMC4705679

[B85] Simabuco F. M. Tamura R. E. Pavan I. C. B. Morale M. G. Ventura A. M. (2020). Molecular mechanisms and pharmacological interventions in the replication cycle of human coronaviruses. Genet. Mol. Biol. 44, e20200212. 10.1590/1678-4685-GMB-2020-0212 33237152 PMC7731901

[B86] Smith D. F. Galkina E. Ley K. Huo Y. (2005). GRO family chemokines are specialized for monocyte arrest from flow. Am. J. Physiol. Heart Circ. Physiol. 289, H1976–H1984. 10.1152/ajpheart.00153.2005 15937099

[B87] Smyth D. J. Plagnol V. Walker N. M. Cooper J. D. Downes K. Yang J. H. M. (2008). Shared and distinct genetic variants in type 1 diabetes and celiac disease. N. Engl. J. Med. 359, 2767–2777. 10.1056/NEJMoa0807917 19073967 PMC2840835

[B88] Su M. Chen Y. Qi S. Shi D. Feng L. Sun D. (2020). A mini-review on cell cycle regulation of coronavirus infection. Front. Vet. Sci. 7, 586826. 10.3389/fvets.2020.586826 33251267 PMC7674852

[B89] Suomi T. Seyednasrollah F. Jaakkola M. K. Faux T. Elo L. L. (2017). Rots: An R package for reproducibility-optimized statistical testing. PLoS Comput. Biol. 13, e1005562. 10.1371/journal.pcbi.1005562 28542205 PMC5470739

[B90] Tepasse P-R. Vollenberg R. Steinebrey N. König S. (2022). High angiotensin-converting enzyme and low carboxypeptidase N serum activity correlate with disease severity in COVID-19 patients. J. Pers. Med. 12, 406. 10.3390/jpm12030406 35330406 PMC8949860

[B91] Tzilas V. Manali E. Papiris S. Bouros D. (2020). Intravenous immunoglobulin for the treatment of COVID-19: A promising tool. Respiration; international review of thoracic diseases, 1087–1089. 10.1159/000512727 PMC7801989 33212437

[B92] van Vollenhoven R. F. Bieber M. M. Powell M. J. Gupta P. K. Bhat N. M. Richards K. L. (1999). VH4-34 encoded antibodies in systemic lupus erythematosus: A specific diagnostic marker that correlates with clinical disease characteristics. J. Rheumatol. 26, 1727–1733. Available: https://www.ncbi.nlm.nih.gov/pubmed/10451069 . 10451069

[B93] Verity R. Okell L. C. Dorigatti I. Winskill P. Whittaker C. Imai N. (2020). Estimates of the severity of coronavirus disease 2019: A model-based analysis. Lancet. Infect. Dis. 20, 669–677. 10.1016/S1473-3099(20)30243-7 32240634 PMC7158570

[B94] Voora D. Cyr D. Lucas J. Chi J-T. Dungan J. McCaffrey T. A. (2013). Aspirin exposure reveals novel genes associated with platelet function and cardiovascular events. J. Am. Coll. Cardiol. 62, 1267–1276. 10.1016/j.jacc.2013.05.073 23831034 PMC3786046

[B95] Wall G. C. Smith H. L. Trump M. W. Mohr J. D. DuMontier S. P. Sabates B. L. (2021). Pentoxifylline or theophylline use in hospitalized COVID-19 patients requiring oxygen support. Clin. Respir. J. 15, 843–846. 10.1111/crj.13363 33735520 PMC8250682

[B96] Wang P-G. Tang D-J. Hua Z. Wang Z. (2020). Sunitinib reduces the infection of SARS-CoV, MERS-CoV and SARS-CoV-2 partially by inhibiting AP2M1 phosphorylation. Cell. Discov. 6, 71. 10.1038/s41421-020-00217-2 33083006 PMC7550610

[B97] Warszawska J. M. Gawish R. Sharif O. Sigel S. Doninger B. Lakovits K. (2013). Lipocalin 2 deactivates macrophages and worsens pneumococcal pneumonia outcomes. J. Clin. Investig. 123, 3363–3372. 10.1172/JCI67911 23863624 PMC3726165

[B98] Wilk A. J. Rustagi A. Zhao N. Q. Roque J. Martínez-Colón G. J. McKechnie J. L. (2020). A single-cell atlas of the peripheral immune response in patients with severe COVID-19. Nat. Med. 26, 1070–1076. 10.1038/s41591-020-0944-y 32514174 PMC7382903

[B99] Witvrouw M. Daelemans D. Pannecouque C. Neyts J. Andrei G. Snoeck R. (1998). Broad-spectrum antiviral activity and mechanism of antiviral action of the fluoroquinolone derivative K-12. Antivir. Chem. Chemother. 9, 403–411. 10.1177/095632029800900504 9875393

[B100] Wu Z. McGoogan J. M. (2020). Characteristics of and important lessons from the coronavirus disease 2019 (COVID-19) outbreak in China: Summary of a report of 72 314 cases from the Chinese center for disease control and prevention. JAMA 323, 1239–1242. 10.1001/jama.2020.2648 32091533

[B101] Xu Y-P. Qiu Y. Zhang B. Chen G. Chen Q. Wang M. (2019). Zika virus infection induces RNAi-mediated antiviral immunity in human neural progenitors and brain organoids. Cell. Res. 29, 265–273. 10.1038/s41422-019-0152-9 30814679 PMC6461993

[B102] Yang X. Yu Y. Xu J. Shu H. Xia J ’an Liu H. (2020). Clinical course and outcomes of critically ill patients with SARS-CoV-2 pneumonia in wuhan, China: A single-centered, retrospective, observational study. Lancet. Respir. Med. 8, 475–481. 10.1016/S2213-2600(20)30079-5 32105632 PMC7102538

[B103] Zaid Y. Guessous F. Puhm F. Elhamdani W. Chentoufi L. Morris A. C. (2021). Platelet reactivity to thrombin differs between patients with COVID-19 and those with ARDS unrelated to COVID-19. Blood Adv. 5, 635–639. 10.1182/bloodadvances.2020003513 33560374 PMC7846461

[B104] Zhang J-Y. Wang X-M. Xing X. Xu Z. Zhang C. Song J-W. (2020). Single-cell landscape of immunological responses in patients with COVID-19. Nat. Immunol. 21, 1107–1118. 10.1038/s41590-020-0762-x 32788748

[B105] Zhang Q. Zhang C. Xi Z. (2008). Enhancement of RNAi by a small molecule antibiotic enoxacin. Cell. Res. 18, 1077–1079. 10.1038/cr.2008.287 18813225

[B106] Zhang Y-N. Zhang Q-Y. Li X-D. Xiong J. Xiao S-Q. Wang Z. (2020). Gemcitabine, lycorine and oxysophoridine inhibit novel coronavirus (SARS-CoV-2) in cell culture. Emerg. Microbes Infect. 9, 1170–1173. 10.1080/22221751.2020.1772676 32432977 PMC7448857

[B107] Zhou Y. Zhou B. Pache L. Chang M. Khodabakhshi A. H. Tanaseichuk O. (2019). Metascape provides a biologist-oriented resource for the analysis of systems-level datasets. Nat. Commun. 10, 1523. 10.1038/s41467-019-09234-6 30944313 PMC6447622

[B108] Zucoloto A. Z. Jenne C. N. (2019). Platelet-neutrophil interplay: Insights into neutrophil extracellular trap (NET)-Driven coagulation in infection. Front. Cardiovasc. Med. 6, 85. 10.3389/fcvm.2019.00085 31281822 PMC6595231

